# Beyond the T: Volumetric MRI Predicts Lymphatic Spread in Oral Squamous Cell Carcinoma

**DOI:** 10.3390/cancers18040692

**Published:** 2026-02-20

**Authors:** Philipp Thoenissen, Davide Giardino, Ibrahim Yel, Thomas J. Vogl, Scherwin Mahmoudi, Andreea I. Nica, Julian Diers, Max Fleischmann, Christian Issing, Robert Sader, Rossano Girometti, Tommaso D’angelo, Christian Booz, Shahram Ghanaati

**Affiliations:** 1 Department of Oral, Cranio-Maxillofacial and Plastic Facial Surgery, Goethe University Frankfurt, 60596 Frankfurt am Main, Germany; thoenissen@med.uni-frankfurt.de (P.T.); julian.diers@ukffm.de (J.D.); sader@em.uni-frankfurt.de (R.S.); s.ghanaati@med.uni-frankfurt.de (S.G.); 2 Department of Radiology and Nuclearmedicine, Goethe University Frankfurt, 60596 Frankfurt am Main, Germany; ibrahim.yel@unimedizin-ffm.de (I.Y.); t.vogl@em.uni-frankfurt.de (T.J.V.); andrea-ioana.nica@unimedizin-ffm.de (A.I.N.); christian.booz@unimedizin-ffm.de (C.B.); 3 Department of Radiotherapy and Oncology, Goethe University Frankfurt, 60596 Frankfurt am Main, Germany; 4 Department of Otorhinolaryngology, Goethe University Frankfurt, 60596 Frankfurt am Main, Germany; 5 Institute of Radiology, Department of Medicine, University of Udine, University Hospital “S. Maria della Misericordia”, ASUFC, 33100 Udine, Italy; rossano.girometti@uniud.it; 6 Diagnostic and Interventional Radiology Unit, BIOMORF Department, University Hospital Messina, 98100 Messina, Italy; tdangelo@unime.it

**Keywords:** oral squamous cell carcinoma, tumor volume, MRI, cervical lymph nodes, metastatic spread, neck dissection

## Abstract

Purpose: To assess the relationship between primary tumor volume, the spatial distribution of cervical lymph node metastases, and TNM (Tumor, node, metastasis) staging in patients with oral squamous cell carcinoma (OSCC) based on high-resolution MRI. Methods: This retrospective analysis evaluated 116 predominantly male (62.9%) patients (age 67.7 ± 11.5 years) with histologically confirmed OSCC who underwent surgical resection with neck dissection or definitive chemoradiation. MRI-based volumetry and center-to-center distance measurements between primary tumor and cervical lymph node metastases were performed using 3D postprocessing software. Results: Tumor-to-lymph node center-to-center distances ranged from 11.7 to 117.3 mm (median: 44.8 mm; interquartile range [IQR]: 32.1–59.6 mm). Primary tumor volume ranged from 0.5 to 87.2 cm^3^ (median: 13.3 cm^3^; IQR: 6.4–21.5 cm^3^) and was higher in male patients. A significant association was observed between tumor volume and both lymph node distance and pT-stage (*p* < 0.0001). The association between tumor volume and tumor–node distance was stronger (Spearman’s r = 0.4541, *p* < 0.0001) than that between pT-stage (TNM classification) and nodal distance (Spearman’s r coefficient =0.2682, *p* = 0.0036). Conclusion: MRI-based assessment indicated that tumor volume has a stronger association with the spatial extent of lymph node metastases compared with T-stage alone. Larger tumors were associated with greater distances to metastatic lymph nodes. These findings highlight the value of quantitative 3D MRI-based volume and distance analyses in potentially improving surgical and radiotherapeutic planning.

## Simple Summary

In this retrospective study, we investigate the relationship between primary tumor volume, the spatial distribution of cervical lymph node metastases, and TNM staging in patients with oral squamous cell carcinoma. Using high-resolution MRI-based 3D volumetric analysis and center-to-center distance measurements, we quantitatively assessed metastatic spread patterns in a cohort of 116 patients treated with either primary surgery and neck dissection or definitive chemoradiation. Our results demonstrate that tumor volume shows a significantly stronger association with the spatial extent of lymph node metastases than T-stage alone. Larger primary tumors were associated with greater distances to metastatic lymph nodes and a higher likelihood of bilateral nodal involvement. The significance of this work lies in its potential to improve individualized surgical and radiotherapeutic planning in OSCC.

## 1. Introduction

Head and neck cancer is represented by squamous cell carcinoma and involves different regions in that area. Among head and neck malignancies, oral cancer is the most common subtype, with squamous cell carcinoma (OSCC) representing the predominant histological entity. While it may arise at multiple oral sites, it most frequently affects the tongue and the floor of the mouth [1,2], which together account for approximately 90% of all oral cavity malignancies [3]. The most important prognostic factor in squamous cell carcinoma of the head and neck depends upon the presence of metastatic disease in the regional lymph nodes [4]. Therefore, identification of cervical metastatic lesions is crucial for physicians hoping to devise a comprehensive therapeutic plan. Diagnosis of cervical lymph node metastases has long been predominantly performed physically. Palpation of the neck, however, even by experienced hands, is not uniformly reliable in the assessment of regional metastatic disease [5]. Magnetic resonance imaging (MRI) and computed tomography (CT) are used by physicians for determining the presence of cervical lesions [6,7]. MRI has potential advantages over CT, including detection of perineural spread, vascular involvement, and invasion of adjacent structures, as well as better soft tissue contrast [8,9,10,11,12]. In addition, diffusion-weighted MRI imaging has been investigated as a method of increasing specificity for the detection of nodal metastases and may add further benefit [13]. Also other diagnostic modalities like Positron-Emission-Tomography in combination with CT and ultrasound play an important role in locoregional staging of OSCC. Previously, tumor volume has been evaluated as a prognostic factor in OSCC tumor volume [14]. Higher tumor volumes are reported to give poorer overall survival rates in OSCC [15,16].

The novelty our study is the combined implementation of 3D MRI-based volumetry with a continuous center-to-center tumor–node distance metric.

We aim to investigate whether there is a correlation between the volume of the primary tumor and the distance of lymph node metastases, as well as with the volume of lymph node metastases and the TNM-classification in patients with OSCC. Our idea provides not only qualitative but also quantitative information through the calculation of volume and distance, as well as a 3D representation of the tumor volume and metastases, which can assist the surgeon and therapist during surgical planning, the operation itself and planning and administration of radiotherapy.

## 2. Materials and Methods

Approval for this retrospective analysis was given by the local ethics committee. In this retrospective study, 116 consecutive patients (m: *n* = 73, 63%, f: *n* = 43, 37%) with OSCC who underwent primary tumor resection and/or chemoradiation of the University Hospital Frankfurt between 1 January 2013 and 20 May 2022 were evaluated. Patients’ mean age was 67.8 years (range 31–101 years; SD ± 11.45 years).

**Figure 1:**
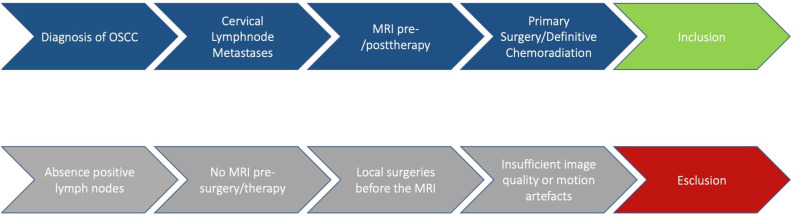
Description of the workflow.

All patients were consecutively identified from the institutional database, but inclusion in the final analysis required the presence of radiologically evident cervical lymph node metastases. The following inclusion criteria were applied: primary resection of oral and pharyngeal cancer with neck dissection, definitive chemoradiation, with mandatory pre-treatment MRI, and radiological evidence of nodal disease. (Figure 1).

The exclusion criteria were the absence of positive lymph nodes, the absence of MRI before surgery/therapy and/or the performance of local surgeries before the MRI assessment, and insufficient radiological imaging with motion artefacts. Approximately 90 patients (43.7%) were excluded from the study because they did not meet the inclusion criteria. In particular, these patients were excluded at the beginning of the recruitment process because they had not undergone MRI, or because the MRI examinations were affected by motion artifacts or susceptibility artifacts due to the presence of dental prostheses.

The evaluated parameters comprised tumor stage according to the TNM classification (tumor, node, metastasis), along with patient sex and age. Tumor staging was performed following the guidelines of the American Joint Committee on Cancer (AJCC) and corresponding national guidelines [17,18].

### 2.1. Primary Surgery

Patients eligible for primary surgery received tumor resection and a combination of neck dissection as single side modified radical neck dissection comprising 5 levels and/or expansion to the contralateral side. Reconstruction following tumor resection was performed using local flaps or microsurgically assisted free flap techniques, depending on the extent of the defect. Based on individual risk factors, a postoperative risk-adapted chemoradiation protocol was applied.

### 2.2. Definitive Chemoradiation

For definitive chemoradiation (CRT), radiotherapy was delivered to the primary tumor (70.6 Gy), involved lymph nodes (59.4–70.6 Gy), high-risk nodal levels (59.4 Gy), and elective neck levels (49.6 Gy), five days per week. Patients initially received 30 × 2 Gy, followed by hyperfractionated accelerated radiotherapy up to a total dose of 40.6 Gy delivered as 1.4 Gy twice daily (BID). To define boost volumes, a re-staging CT scan was performed between 45 and 50 Gy. Concurrent platinum-based chemotherapy was administered as a split course on days 1–5 and 29–34, consisting of cisplatin (20 mg/m^2^ BSA) or carboplatin for cisplatin-ineligible patients (AUC 1), in combination with 5-fluorouracil (600 mg/m^2^ BSA). Clinical follow-up, including panendoscopy and imaging, was scheduled 12 weeks after completion of CRT.

### 2.3. Data Acquisition

MRI was acquired for all patients with a field strength of 3 Tesla (Siemens Magnetom Prisma Fit, Siemens Healthineers, Forchheim, Germany). Although a full multiparametric MRI protocol was available for all patients, including contrast-enhanced T1-weighted imaging, high-resolution T2-weighted sequences, and diffusion-weighted imaging, volumetric segmentation and distance measurements were deliberately performed using a single T1-weighted TSE fat-suppressed Dixon sequence (T1 TSE fs dixon). This approach was chosen to ensure geometric consistency and reproducibility of three-dimensional measurements across patients. While complementary sequences are known to improve nodal characterization and diagnostic confidence, particularly for differentiating benign from malignant lymph nodes, their integration into volumetric workflows may introduce variability related to spatial resolution, distortion, and sequence-specific contrast behavior. T1 TSE fs dixon was acquired in both axial and coronal orientations at equal slice thickness (Table 1).

Imaging was planned to include the thoracic-cervical junction up to the skull base. If severe motion artifacts occurred, image sequences were repeated up to two times. The best image series was included if the repeated acquisition was performed. Patients with examinations of insufficient image quality were excluded from the analysis.

### 2.4. Postprocessing of Data

Volumetry was performed on a SyngoVia (Version VB10B, Siemens Healthcare). The segmentation and calculation of the volume of the primary tumor and lymph node metastases were performed; subsequently, the distance between these two was calculated using the central point as a reference. The distance is defined as the Euclidean center-of-mass-to-center-of-mass distance between the primary tumor volume and the metastatic lymph node. All analyses were conducted at the patient level. In patients with multiple metastatic nodes, the maximum tumor–node distance was used, reflecting the clinically relevant spatial extent of nodal spread.

**Figure 2:**
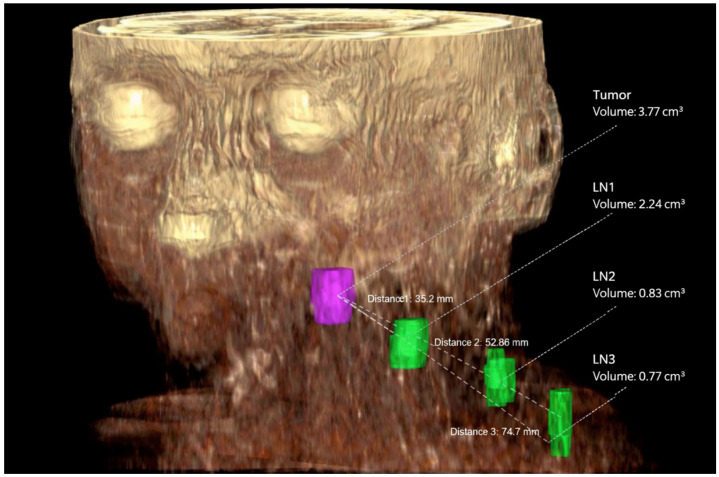
3D representation of the tumor volume (purple) and three lymph node metastases (green) at levels IIa, IVa, and IVb. The tumor volume is 3.77 cm^3^, and the lymph node metastasis volumes are 2.24 cm^3^, 0.83 cm^3^, and 0.77 cm^3^, respectively. The distances D1 between the tumor volume and the first lymph node metastasis (35.2 mm), D2 (52.86 mm), and D3 (74.7 mm) are also reported.

Volumetric segmentation was performed manually, following standardized anatomical boundaries. All segmentations were initially performed by a radiology resident with five years of training and experience and were subsequently reviewed and approved by a board-certified radiologist with dedicated expertise in head and neck imaging. Volumes are reported in cm^3^. 3D volumetry was checked in 3D segmentation mode (Figure 2).

### 2.5. Statistical Analysis

Statistical analysis was performed using commercially available software (GraphPad Prism, version 10.0). Categorical variables are presented as absolute numbers and percentages. Continuous variables are reported as mean ± standard deviation (SD) for normally distributed data and as median with interquartile range (IQR) for non-normally distributed data. Normality was assessed using the Shapiro–Wilk test. Group comparisons (sex, nodal metastases, T-stage groups) for non-normally distributed continuous variables were performed using non-parametric tests. Associations between continuous variables were evaluated using Spearman’s rank correlation coefficient. A *p* value < 0.05 was considered statistically significant.

## 3. Results

### 3.1. General Findings

A total of 116 patients were included in the study. Of these, 74 patients (63.8%) underwent primary tumor resection with unilateral or bilateral neck dissection, while 42 patients (36.2%) were treated with definitive chemoradiation (CRT) without undergoing any surgery or lymph node dissection. For patients treated with CRT, histopathological confirmation of nodal status was not available; therefore, cervical lymph node metastases were defined based on established MRI criteria (including nodal necrosis, contrast enhancement, and diffusion restriction), complemented by clinical staging and follow-up response assessment. For surgically treated patients, histopathology served as the reference standard.

Most patients were male (*n* = 73, 62.9%). Patient age ranged from 31 to 101 years, with a mean age of 67.7 ± 11.5 years. The mean age was 67 years in the surgical group and 69 years in the CRT group. The most common primary tumor site was the oral cavity (*n* = 75, 64.7%), followed by the oropharynx (*n* = 33, 28.4%) and hypopharynx (*n* = 8, 6.9%). Regarding disease stage, 17 patients (14.6%) presented with stage I disease, while 34 (29.3%), 37 (32.0%), and 28 (24.1%) patients had stage II, III, and IV disease, respectively.

### 3.2. Distance Tumor-Lymph Node

**Figure 3:**
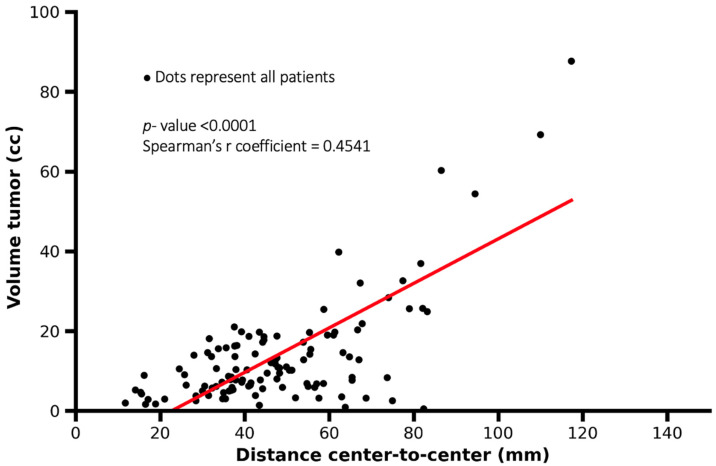
Scatter plot showing the association between tumor volume and tumor–lymph node center-to-center distance. Each dot represents one patient. Larger tumor volumes were associated with greater tumor–lymph node distances, although substantial variability was observed (Spearman’s r = 0.4541, *p* < 0.0001).

Based on MRI measurements, the tumor–lymph node center-to-center distance ranged from 11.7 to 117.3 mm, with a median distance of 44.8 mm (interquartile range [IQR]: 32.1–59.6 mm). A significant association was observed between primary tumor volume and tumor–lymph node distance (Spearman’s r coefficient = 0.4541, *p* < 0.0001), indicating that larger tumor volumes were associated with greater spatial distances to metastatic lymph nodes (Figure 3).

**Figure 4:**
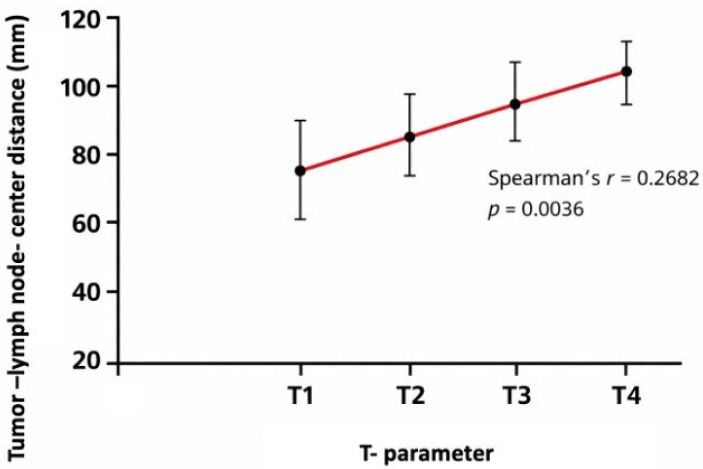
Mean tumor–lymph node center-to-center distance according to T category (TNM classification). Distances were calculated as the maximum tumor–node center-to-center distance per patient. Error bars indicate standard deviation. Mean values per T category were: T1 = 74.9 mm, T2 = 83.2 mm, T3 = 94.5 mm, and T4 = 117.3 mm (Spearman’s r = 0.2682, *p* = 0.0036).

A significant correlation also exists between the distance of lymph node metastases and the T-parameter (TNM classification) (*p* = 0.0036, Spearman’s r coefficient= 0.2682); therefore, higher T categories were associated with greater tumor–lymph node center-to-center distances (Figure 4). According to the study results, the distance of lymph node metastases was greater in male patients compared to female patients (*p* < 0.0001).

### 3.3. Distances and Volumes

**Figure 5:**
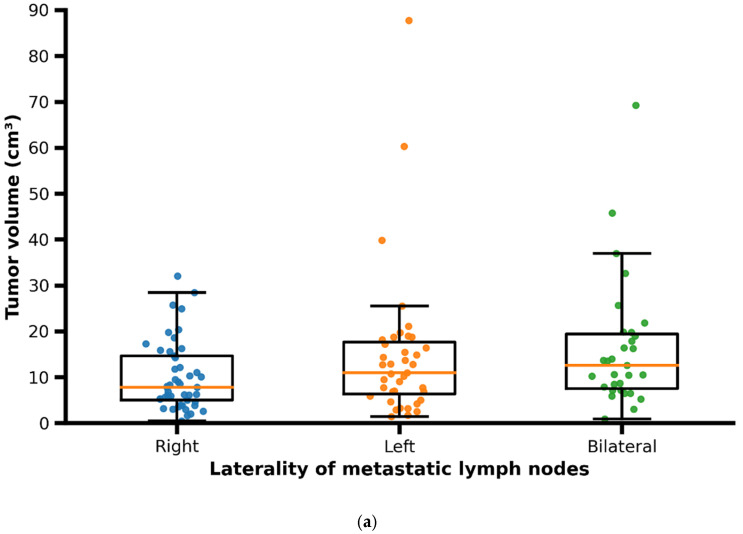

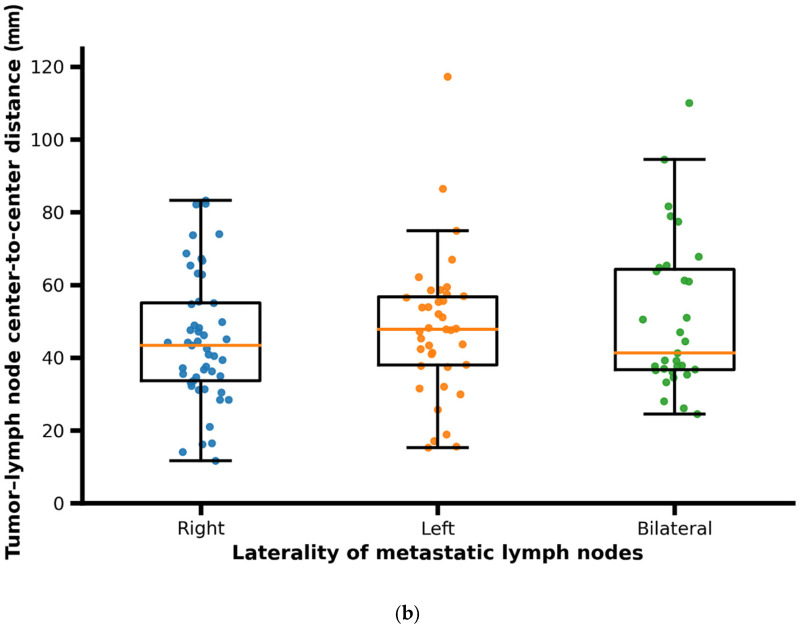
(**a**) Scatter/box plot of primary tumor volume according to laterality of metastatic lymph nodes (right, left, bilateral). Each dot represents one patient; boxes indicate median and interquartile range. In patients with bilateral metastases, distances correspond to the maximum tumor–lymph node center-to-center distance per patient. (**b**) Scatter/box plot of tumor–lymph node center-to-center distance according to laterality of metastatic lymph nodes (right, left, bilateral). Each dot represents one patient; boxes indicate median and interquartile range. In patients with bilateral metastases, distances correspond to the maximum tumor–lymph node center-to-center distance per patient.

The primary tumor volume ranged from 0.5 to 87.2 cm^3^, with a median of 13.3 cm^3^ (interquartile range [IQR]: 6.4–21.5 cm^3^). Median tumor volume was higher in male patients compared to female patients. The volume of metastatic lymph nodes ranged from 0.3 to 36.7 cm^3^, with a median of 4.45 cm^3^ (IQR: 2.1–8.0 cm^3^). Patients presenting with bilateral cervical lymph node metastases showed larger primary tumor volumes and greater tumor–lymph node center-to-center distances compared with patients with unilateral (right- or left-sided) nodal metastases (Figure 5a,b).

### 3.4. Side of Metastases

A total of 30 patients (25.9%) had a tumor that metastasized to both sides with a tumor volume in these patients, that ranged from 0.9 to 69.2 cm^3^ with a median of 16.3 cm^3^ (±14.2). In 48 patients (41.4%) the tumor metastasized to right side; in those patients the tumor volume ranged from 0.5 to 32.1 cm^3^ with a median of 10.5 cm^3^ (±7.5). A total of 38 patients (32.8%) showed lymph node metastases on the left side; the volume of tumors in these patients ranged from 1.4 to 87.7 cm^3^ with a median of 14.9 cm^3^ (±16.4).

Additionally, it was observed that bilateral lymph node metastases were not necessarily larger on average compared to unilateral lymph node metastases.

**Figure 6:**
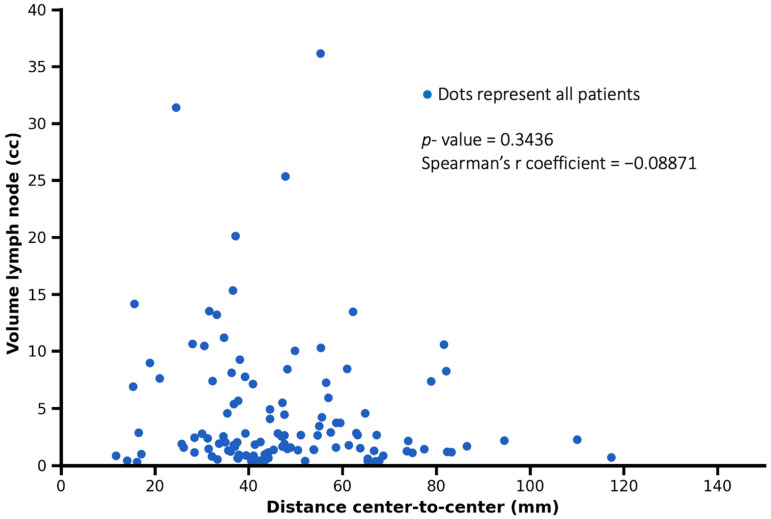
Representation of the relationship between tumor–lymph node center-to-center distance and lymph node metastasis volume. Each dot represents one patient. No significant correlation was observed (Spearman’s r = −0.08871, *p* = 0.3436).

No significant correlation was observed between the volume of lymph node metastases and the distance from the primary tumor (Spearman’s r = −0.0887, *p* = 0.3436). This finding suggests that lymph node size was not associated with tumor-to-node distance, with smaller and larger metastatic lymph nodes occurring across a similar range of distances (Figure 6).

## 4. Discussion

This study evaluated tumor volume and the correlation between tumor volume and cervical lymph node metastases in 116 consecutive patients undergoing primary surgery with neck dissection or definitive chemoradiation in a 3D visualization setting.

Particularly, surgical planning in the field of head and neck oncologic surgery still relies on descriptive–anatomical features and established prognostic factors [17,18]. The cervical dimensions are strictly regulated according to a 35-year-old classification described by Robbins and Medina [19,20,21]. Nevertheless, the actual therapeutical implications are based on these works [22,23]. In addition, 3D visualization and artificial intelligence (AI) are upcoming topics in medicine. They are used in several subentities and parts of planning radiotherapy regimes already [24]. But they do not currently cover a multidisciplinary treatment approach in head and neck oncologic therapy. Deductively, the underlying study highlights the importance of calculating tumor volume, using a 3D resonance software to predict the therapy regime in dependence on tumor volume and spread of lymph node metastases.

In the comparison between TNM classification and volumetric analysis regarding their correlation with the distance between the primary tumor and lymph node metastases, tumor volume demonstrated a significantly stronger association. These findings challenge the adequacy of the T-classification in accurately characterizing the primary tumor. For instance, tumors classified as T4 are defined by invasion into adjacent structures rather than by their actual volume. In contrast, the proposed volumetric classification offers a more nuanced and precise depiction of tumor burden and its relationship with lymphatic spread.

Instead of application of an anatomic–descriptive system, 3D volumetry and distance measurements offer the opportunity to implement an absolute coordinate system. This system grounds digital therapy planning and intraoperative navigation. Earlier, Bittermann et al. already proposed using intraoperative navigation and digital clip marking of frozen sections to increase the accuracy in communication between surgeon and pathologist [25]. The present study relies on this 3D-based workflow that was previously introduced. Given the superior correlation of tumor volume with nodal spread observed in our cohort, future staging systems might benefit from incorporating volumetric parameters as complementary metrics alongside current T-classifications, potentially improving risk stratification and treatment decision-making.

Recently, MRI- or CT-determined tumor volume has been proven as a prognostic factor in OSCC [14,26,27,28]. Patients diagnosed with OSCC and having a tumor volume above 3 cm^3^ have poor outcomes in comparison to patients with smaller tumor volume. Tumor volume has been added to the variety of the established factors like pR-Status, defined as the pathological resection margin status (R0: negative margins; R1/R2: positive margins) [29,30]. Another study stated that using image fusion of MRI and CT in building a digital patient is possible and depiction of a metastatic OSCC disease is feasible [31].

Based on this study, although a significant association between tumor volume and tumor–lymph node center-to-center distance was observed, the correlation strength was moderate rather than strong. As illustrated in Figure 3, small tumor volumes showed a wide distribution of nodal distances, indicating substantial inter-patient variability. In contrast, larger tumor volumes tended to be associated with greater nodal distances, suggesting a general spatial trend rather than a strict predictive relationship. Therefore, tumor volume should be interpreted as an imaging-based indicator associated with nodal spread extent rather than a deterministic predictor at the individual patient level. Patients diagnosed with bilateral cervical metastases also show lymph node metastases at a greater distance to the primary tumor. Additionally, bilateral metastases are more often seen in patients with higher primary tumor volume. This information, both qualitative and quantitative, allows for a better and more precise description of cervical lymph node metastases, aiding the surgeon and radiotherapist during surgical planning and the procedure. With the new imaging techniques, selective surgical planning of a partial resection within levels is feasible disregarding anatomical–descriptive restriction. This image-guided approach must be translated into the surgical and radiotherapeutic field.

Tendentially, surgical therapy comprising neck dissection and radiotherapy must be expanded in the case of higher primary tumor volumes. Also, fields of irradiation must be widened to address all potential lymph node metastases in the neck region. In addition, higher tumor volumes hint a bilateral neck dissection or irradiation planning including both sides of the neck area. These implications are also reflected in the guidelines [32]. The guidelines already rely on tumor-specific factors, i.e., entity of tumor, crossing of midline and positive frozen sections. However, they show important value due to the new findings in this study. Although the conventional rationale for widening the therapeutic field is to ensure comprehensive disease control, this approach may inadvertently increase the risk of treatment-related morbidity [33]. Consequently, indiscriminate expansion of the surgical or radiotherapeutic margins is not recommended. Instead, a selective strategy targeting specific lymph nodes –potentially extending beyond traditional anatomical levels—may offer a more balanced risk–benefit profile. Image-guided techniques enable precise localization and targeted dissection of lymph nodes, including those at greater spatial distances from the primary tumor, thereby preserving oncological efficacy while minimizing unnecessary exposure to surrounding tissue.

However, the malignancy of lymph node metastases does not depend on their volume in general [34]; small metastases can be found on both cervical sides, which underlines the importance of the elective (diagnostic) ND along with the knowledge about occult cervical metastases [35].

Our study showed that male patients have lymph node metastases at a greater distance. Also, male patients are shown to have higher tumor volumes in our study. Concerning German governmental data, men are affected by OSCC twice as frequently as women and diagnosed with higher UICC stages according to the Union for International Cancer Control (UICC) classification: 42% of men have primary diagnoses in UICC-stage IV, whereas 37% of women are primarily diagnosed with UICC-stage IV [36].

Up-to-date MRI and CT diagnostic imaging might be associated with a high accuracy in detecting lymph node metastases and analyzing tumor infiltration depth [7]. A diameter greater than 1 cm, central necrosis and contrast enhancement are signs of malignancy but not evidence [37]. Also, PET/CT, as an additional tool, might be considered to enhance the accuracy [38]. While PET/CT remains the gold standard for metabolic assessment, it may not provide the same spatial resolution or anatomical detail as high-resolution MRI. The integration of volumetric MRI with PET-derived functional metrics could yield synergistic models for more accurate prediction of lymphatic dissemination. In addition, the integration of precise volumetric and spatial data into radiotherapy planning could allow for patient-specific expansion margins, potentially improving coverage of microscopic disease while minimizing the dose to adjacent critical structures.

The distances between primary tumor and lymph node metastases did not differ significantly when comparing the surgery and chemoradiation group. Although cervical staging may be less precise in non-surgically treated patients, the combination of clinical and radiological assessment represents an accepted surrogate for histopathological evaluation, which remains the gold standard in surgically treated patients.

In contrast to all the benefits of the described workflow, there are limitations. A relevant limitation of the present study is the inclusion of patients treated with definitive chemoradiotherapy, for whom histopathological confirmation of cervical lymph node metastases is not available. In this subgroup, nodal involvement was determined based on pre-treatment imaging and clinical staging, which may introduce a risk of nodal misclassification. In particular, imaging-based criteria may overestimate nodal disease due to limited specificity, potentially leading to false-positive classification of metastatic lymph nodes.

However, the primary aim of this study was not to validate imaging findings against histopathology on a node-by-node basis, but to investigate imaging-derived spatial relationships between primary tumor volume and nodal distribution as assessed on pre-treatment MRI, reflecting real-world clinical decision-making. Importantly, sensitivity analyses restricted to surgically treated patients with histopathological confirmation demonstrated comparable association trends, supporting the robustness of the main findings. Patients without cervical lymph node metastases were not included in the present analysis, as the study focused on spatial characteristics of nodal spread in N+ disease. Future studies including both N0 and N+ patients may further clarify the relationship between tumor volume and the likelihood of nodal involvement.

The applied approach is time consuming in preparation. Volumetry, distance measurements, and the proper segmentation of the tumor and lymph nodes, which reflect the patient’s status, take up to one hour and may also prolong the demonstration in an oncologic board meeting. This work might be shortened using internal software tools and AI capacity focusing on automated segmentation. Currently, volumetric segmentation and distance measurements require dedicated software and trained personnel, limiting widespread implementation. However, the rapid evolution of AI-based tools may soon enable fully automated, real-time 3D analysis, reducing workload and increasing scalability. Additionally, this study covers patients treated with surgery and chemoradiation by different departments, all data was collected in a single center and only patients undergoing surgery had histopathological proof of lymph node metastases.

The 3D visualization and distance measurements require a full 3D segmentation of the patient’s disease. In this dimension, the work of the preparing physician leads to a 3D model which can assist the surgeon and radiotherapist in understanding and planning the proper individual therapy regimen. Students, trainees and junior doctors without experienced clinical knowledge may benefit from the 3D depiction [39]. The 3D visualization should be established as a standard in the case of discussions within the multidisciplinary oncologic board meetings.

Beyond surgical and radiotherapeutic planning, it remains to be further investigated to which extent primary tumor volume also bears prognostic relevance for disease-specific or overall survival. Future longitudinal studies are needed to explore volumetry as a biomarker in outcome prediction.

## 5. Conclusions

In conclusion, the results of this study support the routine incorporation of 3D volumetric and spatial tumor analysis in OSCC management. Tumor volume, more than T-category, is predictive of nodal spread distance and pattern. These data enable a more individualized, precise approach to neck dissection and radiation planning, potentially improving oncologic outcomes while preserving function and quality of life.

The general approach of assessing lymph node levels according to the Medina Classification must be questioned.

## Figures and Tables

**Table 1:** Overview of MRI details of the protocol for sequences using T1-weighted in axial orientation.

MRI Protocol	T1-Weighted Axial
MRI scanner model	Magnetom Prisma Fit
Sequence type	T1-weighted TSE fs dixon
Repetition time	659 milliseconds
Echo time	12 milliseconds
Orientation	Transverse
Coding direction	Anterior–posterior
Slice thickness	4 mm
Distance factor	20%
Voxel size	0.7 × 0.7 × 4.0 mm
Field of view	270 × 270 mm
Concatenations	2
Fat suppression	Yes

## Data Availability

Data available in a publicly accessible repository.
